# Analytical Validation and Preliminary Clinical Evaluation of Plasma Carboxylesterase 1 for Ultrasound-Defined Hepatic Steatosis

**DOI:** 10.3390/diagnostics16162658

**Published:** 2026-08-20

**Authors:** Makito Tanaka, Shingo Tanaka, Kayoko Ochi, Ryo Kobayashi, Ryosei Murai, Satoshi Takahashi

**Affiliations:** 1 Division of Laboratory Medicine, Sapporo Medical University Hospital, Sapporo 060-8543, Japan; mkt-tnk@sapmed.ac.jp (M.T.); ochikayo@sapmed.ac.jp (K.O.); r.kobayashi@sapmed.ac.jp (R.K.); r.murai@sapmed.ac.jp (R.M.); stakahas@sapmed.ac.jp (S.T.); 2 Department of Infection Control and Laboratory Medicine, Sapporo Medical University School of Medicine, Sapporo 060-8543, Japan

**Keywords:** attenuation imaging, carboxylesterase 1, enzyme-linked immunosorbent assay, non-invasive liver disease assessment, steatotic liver disease

## Abstract

**Background/Objectives:** Steatotic liver disease (SLD) is a global health priority, increasing the demand for accessible circulating biomarkers. Carboxylesterase 1 (CES1) plays a key role in hepatic lipid metabolism, but its clinical utility as a plasma biomarker remains unclear. This study aimed to validate an automated immunoassay for plasma CES1 and perform a preliminary evaluation of its diagnostic performance for hepatic steatosis quantified using advanced ultrasound. **Methods:** An automated ELISA for plasma CES1 was analytically validated. A preliminary clinical evaluation was performed in 95 consecutive outpatients structured to approximate a health-screening population, using ultrasound-derived attenuation coefficients (AC) to quantify steatosis. **Results:** The assay demonstrated robust analytical performance with intra- and inter-assay coefficients of variation < 10% and excellent sample stability. Plasma CES1 significantly correlated with AC (*r* = 0.585, *p* < 0.001), yielding an area under the receiver operating characteristic curve of 0.82 for diagnosing ultrasound-defined steatotic livers. In multivariable analysis, CES1 was independently associated with hepatic steatosis (adjusted odds ratio: 1.03 per 10 pg/mL increase, *p* < 0.001). **Conclusions:** Plasma CES1 shows strong diagnostic potential for ultrasound-defined hepatic steatosis. However, this preliminary evaluation is subject to a restricted cohort spectrum; further prospective validation in broader, unselected real-world populations is required before clinical implementation.

## 1. Introduction

Steatotic liver disease (SLD) has emerged as a major global health challenge and is estimated to affect nearly one-third of the world’s population. The rising prevalence of SLD is driven primarily by the increasing obesity epidemic and incidence of type 2 diabetes [1]. Recently, the clinical community revised the nomenclature of nonalcoholic fatty liver disease (NAFLD) to metabolic dysfunction-associated steatotic liver disease (MASLD) to better reflect its pathophysiological origins [2,3]. Despite these changes in terminology, identifying hepatic steatosis remains a fundamental first step in clinical management [2].

Historically, hepatic fibrosis has been considered the primary determinant of the long-term prognosis of NAFLD [4,5]. However, contemporary data indicate that the extent of fat accumulation significantly affects clinical outcomes and disease progression [6,7]. Consequently, precise quantification of hepatic steatosis has gained clinical importance. While conventional abdominal ultrasonography (US) is the most widely used screening tool, new quantitative attenuation-based techniques allow for a more objective assessment [8]. However, the logistical demands and costs associated with these advanced imaging modalities limit their feasibility for mass screening. This gap underscores the urgent need for simple, cost-effective blood-based biomarkers.

The mammalian carboxylesterase (CES) family is a multigene superfamily characterized by the α/β-hydrolase fold, comprising 6 human and 20 murine paralogs [9]. These enzymes catalyze the hydrolysis of various xenobiotics and endogenous lipid esters. Within this family, human CES1 is a 62 kDa protein primarily synthesized in the liver [10]. In addition to its role in xenobiotic metabolism, CES1 facilitates the hydrolysis of triglycerides and cholesterol esters. In murine models, the hepatic overexpression of human CES1 mitigates steatohepatitis and atherosclerosis by reducing lipid deposition, oxidative stress, and inflammatory responses [11]. However, the exact contribution of CES1 to lipid homeostasis remains controversial because of the inconsistent findings regarding its effect on cholesterol metabolism [12].

Although preliminary clinical and proteomic evidence has linked CES1 to SLD pathology [13,14,15,16], the relationship between plasma CES1 levels and objective, ultrasound-based fat quantification has not been elucidated. To address this, we established a measurement system for circulating CES1 using a fully automated platform and characterized its analytical and pre-analytical performance. Furthermore, we evaluated its utility as a non-invasive biomarker for hepatic steatosis, aiming to clarify the clinical relevance of plasma CES1 within the diagnostic framework of steatotic liver.

## 2. Materials and Methods

### 2.1. Analytical Validation of CES1 Immunoassay

#### 2.1.1. Automated CES1 Enzyme-Linked Immunosorbent Assay (ELISA) Procedure

Plasma CES1 concentrations were measured with an ELISA kit (Human CES1 ELISA Kit; RayBiotech, Norcross, GA, USA) in accordance with the manufacturer’s protocol. Measurements were carried out on an Evolis automated immunoassay analyzer (Bio-Rad, Hercules, CA, USA): an operator loaded the samples and reagents onto the platform, after which the instrument executed the entire assay protocol automatically. The specimens analyzed were residual clinical plasma obtained from lithium heparin-anticoagulated blood and kept in cryotubes at −80 °C until measurement. Analysts were blinded to all clinical information.

To ensure analytical reliability, the automated validation procedures were performed following the established protocol [17] and our previous report [18], with specific adaptations for CES1 measurement.

#### 2.1.2. Intra- and Inter-Assay Precision

For the within-run (intra-assay) evaluation, three predefined CES1 concentration levels were each measured in 20 replicates within one analytical run. Between-day reproducibility (inter-assay) was examined by testing the same three levels in five replicates on five independent days. Imprecision was expressed as the coefficient of variation (CV), and precision was judged acceptable when the CV fell below 15%, in line with conventional analytical criteria [19].

#### 2.1.3. Limit of Blank (LoB), Limit of Detection (LoD), and Lower Limit of Quantification (LLOQ)

The LoB, LoD, and LLOQ of the assay were established in accordance with Clinical and Laboratory Standards Institute (CLSI) EP17 guidelines [20,21]. The LoB and LoD were calculated at a 95% confidence level from 16 blank replicates (sample diluent) and 20 replicates of a low-concentration sample using the following equations:

LoB = Mean_blank_ + 1.645(SD_blank_)

LoD = LoB + 1.645 (SD_low-concentration sample_)

LLOQ was determined by measuring 16 blank replicates; the minimum quantifiable CES1 concentration was then obtained by interpolating the value corresponding to the mean blank signal plus ten standard deviations (SDs) [17]. To determine the LLOQ, three low-concentration samples (2.5, 5.0, and 10.0 pg/mL) were analyzed in triplicate across three independent analytical runs. The acceptance criteria for the LLOQ required both intra- and inter-assay precision (CV) to be ≤15%, with a recovery between 82.5% and 117.5%, in accordance with the acceptable change limit (ACL, 17.5%).

Calibration curves were fitted using a four-parameter logistic (4PL) regression model. Three independent calibration curves (one per analytical run across three days) were evaluated during LLOQ validation. The analytical measuring interval (AMI) was defined as the range from the established LLOQ to the highest standard concentration (10,000 pg/mL).

#### 2.1.4. Dilution Linearity

Linearity over a broad dynamic range was verified by preparing a serial two-fold dilution series (2-fold to 512-fold) of a high-concentration CES1 plasma pool using the sample diluent supplied with the Human CES1 ELISA Kit. Each dilution point was assayed in duplicate within the same analytical run.

To evaluate dilution linearity using a regression-based procedure, linear (*Y* = *aX* + *b*) and polynomial (second- and third-order) regression models were fitted to the replicate-level data. An F-test for non-linearity was performed to determine whether a polynomial model provided a significantly better fit than the linear model. The slope, intercept, 95% confidence intervals (CIs), coefficient of determination (*R*^2^), and individual residuals were calculated. Percentage recovery at each dilution point was determined using the following equation:

Recovery (%) = (Measured concentration/Theoretical concentration) × 100

#### 2.1.5. Spike Recovery Tests

Three plasma specimens covering distinct CES1 concentrations were each divided into four fractions. To yield a 100 μL final reaction volume, 90 μL of patient plasma (sample volume) was combined with 10 μL of either CES1 standard (standard volume) or sample buffer (unspiked baseline). The exact added masses of the CES1 standard were 40, 80, and 100 pg. The theoretical added concentration (pg/mL) was calculated by dividing the added mass by the final reaction volume (0.1 mL), yielding theoretical added concentrations of 400, 800, and 1000 pg/mL, respectively.

The resulting 12 fractions were each quantified in duplicate within a single run. Because the unspiked baseline (neat sample) was prepared with the same dilution factor (90 μL plasma + 10 μL buffer), no further dilution correction was necessary. The acceptance interval for spike recovery was set at 82.5–117.5%, in accordance with the ACL (17.5%). Recovery was expressed as a percentage using the following equation:

Recovery (%) = ([Measured concentration_spiked sample_ − Measured concentration_neat sample_]

/Theoretical concentration_spiked_) × 100

#### 2.1.6. Interference Assessment

To perform a preliminary screening for potential endogenous interferents, a pooled plasma sample (baseline CES1 concentration: 421.1 pg/mL) was spiked with interfering substances using commercial validation kits (Interference Check A Plus and Interference Check RF Plus; Sysmex, Kobe, Japan) according to the manufacturer’s instructions. The evaluated panel included conjugated bilirubin, unconjugated bilirubin, hemoglobin, chyle, and rheumatoid factor (RF).

For each substance, the plasma pool was spiked across a graded series of six concentrations up to maximum tested levels of 20 mg/dL (342 µmol/L) for conjugated and unconjugated bilirubin, 510 mg/dL for hemoglobin, 1420 FTU for chyle, and 500 IU/mL for RF. Single determinations (*n* = 1) were performed at each concentration point as an initial screening assessment. Relative recovery (%) was derived by comparing the measured concentration of each spiked sample to that of an unspiked control sample containing an equivalent volume of vehicle. A deviation within the ACL was prospectively defined as non-significant interference.

#### 2.1.7. Comparison of CES1 Concentrations Among Different Blood Collection Tubes

Blood samples were obtained from 16 patients to assess matrix-related differences in CES1 measurements. In routine clinical care, blood is independently collected into lithium heparin, ethylenediaminetetraacetic acid (EDTA), and serum tubes, rather than being aliquoted from a single specimen. All the samples were handled under identical laboratory procedures and stored at −80 °C before concurrent CES1 analysis using the same ELISA system.

Specifically, all blood samples were obtained under fasting conditions via a single venipuncture, filling the three tubes sequentially within 1 min. The collection order was not governed by a predetermined rule and was varied without systematic bias across participants. Pre-analytical parameters, including serum clotting time, processing delay, and centrifugation conditions, were strictly controlled in accordance with the standard operating procedures of the clinical laboratory.

#### 2.1.8. Sample Stability Evaluation

Sample stability was evaluated using the same three experimental conditions as previously described [18]: (1) freeze–thaw stability; (2) short-term stability at 4 °C and room temperature; and (3) 1-month storage stability. Briefly, plasma samples at three concentration levels were subjected to multiple freeze–thaw cycles while being stored at −80 °C between cycles; separate aliquots were maintained at 4 °C or room temperature for predefined durations; and additional aliquots were stored at −20 °C for 1 month. Following each designated condition, all the samples were subsequently stored at −80 °C until batch analysis. For each condition and time point, a single specimen was assayed in duplicate. Stability was defined as a mean percentage deviation from baseline.

To evaluate the long-term stability of CES1 under actual clinical sample storage conditions (−80 °C), ten clinical plasma samples that had been preserved at −80 °C for more than 1 year after their initial analysis were re-analyzed in duplicate. Replicate-level results were evaluated to distinguish analytical variation (expressed as intra-assay coefficient of variation, %CV) from storage-related changes (expressed as percentage deviation from baseline).

### 2.2. Clinical Validation

#### 2.2.1. Study Population and Selection Criteria

A total of 1709 consecutive outpatients who underwent abdominal US with attenuation imaging (ATI) at Sapporo Medical University Hospital between April 2023 and January 2024 were screened for this study. Of these, 885 patients had same-day blood sampling using lithium-heparin tubes. Patients were included if they met all of the following criteria: an age of 20–69 years together with a body mass index (BMI) below 30 kg/m^2^; a fibrosis-4 (FIB-4) index under 2.67, so as to remove patients at elevated risk of advanced hepatic fibrosis; an alcohol intake within the MASLD threshold [2] (below 210 g/week for men and below 140 g/week for women, equivalent to under 30 g/day and 20 g/day, respectively); a fasting interval of at least 6 h before imaging; and no hepatic disorder apart from SLD, such as viral hepatitis or drug-induced liver injury. Patients with missing clinical or laboratory data were excluded.

Because multiple exclusion criteria and data omissions frequently overlapped in individual patients, exclusions were conducted as a single combined screen, resulting in 790 exclusions. All final 95 analyzed participants successfully completed both ATI examination and plasma CES1 quantification without any technical or quality control failures (e.g., signal within the validated linear range, no significant hemolysis/lipemia interference). The selection process is summarized in the participant flow diagram (Supplementary Figure S1).

#### 2.2.2. Clinical and Laboratory Data

On the examination day, we recorded each patient’s baseline profile, comprising age, sex, height, weight, and habitual alcohol intake. For all the participants, blood tests performed as part of routine clinical care were analyzed in our hospital laboratory to determine aspartate aminotransferase (AST), alanine aminotransferase (ALT), γ-glutamyl transpeptidase (γ-GT), total bilirubin, albumin, and platelet count using standard biochemical methods. Fibrosis (FIB)-4 index was calculated using the following equation [22]:

FIB-4 index = (AST × Age)/(Platelet count × √ALT)

#### 2.2.3. Abdominal US Examination

Ultrasonography was carried out on an Aplio i700 platform (Canon Medical Systems, Otawara, Japan) fitted with a PVI-475BX convex transducer operating at 1–8 MHz. All examinations were conducted by eight trained sonographers with 1–22 years of experience and one hepatologist with approximately 15 years of experience, all of whom are certified members of the Japan Society of Ultrasonics in Medicine and/or the Japanese Society of Sonographers. All ultrasound images were recorded and stored according to a standardized protocol. All operators were blinded to the plasma CES1 assay results.

Targeting the right hepatic lobe through an intercostal window, we acquired ATI and two-dimensional shear-wave elastography (SWE) following the routine B-mode survey. During ATI, a fan-shaped sampling frame was set over the hepatic parenchyma, and a 2 cm × 4 cm measurement region of interest (m-ROI) was defined at the center of the image, kept clear of major vessels and acoustic shadows, with its top edge placed immediately below the multireflection band in accordance with the vendor’s guidance (Supplementary Figure S2) [23]. The SWE acquisition was conducted in accordance with previously validated protocols [24]. Median values of five measurements were recorded for both the attenuation coefficient (AC) via ATI and shear-wave speed via SWE. For quality control of the ATI, a coefficient of determination (*R*^2^) ≥ 0.70 and interquartile range to median ratio (IQR/median) ≤ 15% were required for the measurements to be considered valid. Although intra- and interobserver variability was not directly measured in the current cohort, high reproducibility for ATI has been well documented in previous studies [8].

Hepatic steatosis was additionally evaluated using a semiquantitative B-mode ultrasound scoring system [25,26] that incorporates three principal imaging features: (A) hepatic brightness and hepatorenal echo contrast; (B) deep attenuation; and (C) vessel blurring. Composite scores ranged from 0 to 6. Details of the scoring criteria are provided in Supplementary Table S1. To minimize observer bias and ensure scoring reliability, all B-mode images were evaluated independently by two experienced readers (a sonographer and a hepatologist), both of whom were strictly blinded to the plasma CES1 results. In cases of scoring discrepancies between the two readers, the final composite score was determined by consensus through mutual discussion.

### 2.3. Statistical Analysis

To assess the analytical stability and acceptable deviation of analyte measurements for this biomarker where within-subject biological variability has not yet been established, the ACL was calculated based solely on analytical imprecision ($CV_{a}$), as described previously [27]:

$$\mathrm{ACL}=2.77\times CV_{a}$$

where 2.77 ($1.96\times \sqrt{2}$) represents the 95% confidence threshold for a statistically significant difference between two measurements, and $CV_{a}$ indicates the inter-assay coefficient of variation (6.3% at the medium concentration level). Thus, a mean percentage change greater than the derived ACL (17.5%) was interpreted as a significant deviation lying outside the acceptable analytical range.

Pairwise method comparisons among blood collection matrices (lithium-heparin plasma, EDTA plasma, and serum) were evaluated using Bland–Altman percentage difference plots and Passing–Bablok regression analysis. Bland–Altman percentage analysis was used to determine the mean relative paired bias (%) and 95% limits of agreement (LoA; mean ± 1.96 SD), testing whether the mean relative bias remained within the predefined 17.5% ACL. Passing–Bablok non-parametric regression was conducted to evaluate proportional and constant bias, where 95% confidence intervals (CIs) for the slope including 1.0 and the intercept including 0.0 were interpreted as indicating no significant proportional or constant bias, respectively.

Correlations among continuous variables were quantified with the Spearman rank method. For comparisons among three independent groups (e.g., ultrasound H-categories and exploratory ATI categories), the Kruskal–Wallis test was performed as a global test. When the global test was statistically significant, post hoc all-pairs multiple comparisons were conducted using the Steel–Dwass test to adjust for multiplicity. Group sizes (*n*), exact adjusted p-values (*P*_adj_), and effect sizes were reported. Effect sizes were quantified using eta-squared ($\eta_{H}^{2}$) for Kruskal–Wallis tests and rank correlation coefficient (*r*) for post hoc comparisons.

The discriminative ability of plasma CES1 for detecting ultrasound-defined steatotic liver was assessed by receiver operating characteristic (ROC) curve analysis, and the area under the curve (AUC) was calculated. The optimal cutoff value was determined using Youden’s index. The 95% (CIs) for Spearman’s rank correlation coefficients and AUC were estimated using a bootstrap resampling method with 2500 iterations. Two-sided testing was applied throughout, and *p* < 0.05 was considered statistically significant.

Continuous variables were retained as continuous predictors without artificial dichotomization to prevent information loss and circularity. To identify factors associated with ultrasound-defined steatotic liver, univariable and multivariable logistic regression analyses were performed. Covariates for the multivariable model were selected based on biological and clinical relevance, rather than solely on univariable statistical significance. To avoid model overfitting, the number of multivariable predictors was constrained according to the events per variable (EPV) rule (≥10 events per parameter) based on the 42 ultrasound-defined steatotic outcomes; thus, the final multivariable model included CES1 (per 10 pg/mL increase), BMI, and γ-GT.

Multicollinearity was evaluated using variance inflation factors (VIFs), with values < 5.0 considered acceptable. Model calibration was assessed using the Lack of Fit test (*p* > 0.05 indicating acceptable calibration). Internal model stability and optimism were evaluated using a 1000-sample bootstrap resampling analysis. Odds ratios (ORs) and 95% CIs were reported. p-values were derived from likelihood ratio tests, and *p* < 0.05 was considered statistically significant.

Statistical power and sample size adequacy were evaluated for the primary analyses (α = 0.05, two-tailed). For correlation analysis (*n* = 95), the sample provided > 99% power to detect the observed correlation (*r* = 0.585) and >80% power to detect moderate correlations (*r* ≥ 0.29). For ROC analysis (*n*_1_ = 42 steatotic vs. *n*_2_ = 53 non-steatotic), *n* = 95 yielded > 99% power to detect an AUC of 0.82 relative to the null hypothesis (AUC = 0.50).

All statistical analyses were performed using JMP Student Edition (version 18.2.0; SAS Institute, Cary, NC, USA).

## 3. Results

### 3.1. Assay Validation

#### 3.1.1. Intra- and Inter-Assay Precisions

The precision performance of the CES1 assay is compiled in Table 1. Intra-assay (within-run) precision was high, with CVs of 8.2%, 5.5%, and 3.8% for low-, medium-, and high-concentration levels, respectively, indicating satisfactory reliability.

Inter-assay (between-run) reproducibility was also acceptable, as reflected by CVs of 9.0%, 6.3%, and 7.0% across the same three concentration levels, confirming that assay precision was consistently maintained over multiple days.

#### 3.1.2. LoB, LoD, and LLOQ Verification

The calculated LoB, LoD and LLOQ were 3.11 pg/mL, 4.58 pg/mL and 5.08 pg/mL, respectively. In the multi-run low-concentration assessment, the 5.00 pg/mL sample met all predefined criteria, demonstrating an intra-assay CV of 5.1% to 7.2%, an inter-assay CV of 5.7%, and a mean recovery of 98.0% (bias: −2.0%). The 2.5 pg/mL concentration failed to meet the precision threshold (CV > 15%). Consequently, 5.00 pg/mL was confirmed as the LLOQ, establishing the AMI as 5.00 to 10,000 pg/mL.

#### 3.1.3. Dilution Linearity

Diluted samples demonstrated linearity across the 2-fold to 256-fold dilution range (Supplementary Table S2). Linear regression analysis of the replicate-level data yielded a slope of 1.069 (95% CI: 1.050 to 1.088), an intercept of −30.4 (95% CI: −82.1 to 21.3), and an $R^{2}$ of 0.999. An F-test for non-linearity confirmed the adequacy of the linear model (p>0.05).

Within the 2-fold to 256-fold range, recoveries ranged from 92.9% to 108.9% (Supplementary Table S2), and all deviations remained within the predefined ACL (17.5%). At the 512-fold dilution, high imprecision (CV = 57.9%) and decreased recovery (81.4%) were observed, demonstrating that this dilution falls outside the quantitative range due to low-concentration imprecision. These findings confirm the 2-fold to 256-fold dilution as the validated linear quantitative range for this assay.

#### 3.1.4. Spike Recovery Tests

Tests were conducted across multiple plasma matrices and spike levels, ranging from 88.4 to 107.8%, with low CVs (Supplementary Table S3). These results demonstrate that the plasma matrix did not adversely affect the ability of the assay to accurately quantify CES1.

#### 3.1.5. Interference Assessment

In our initial single-pool screening assessment, none of the tested endogenous substances (conjugated and unconjugated bilirubin up to 20 mg/dL (342 µmol/L), hemoglobin up to 510 mg/dL, intralipid up to 1420 FTU, and rheumatoid factor up to 500 IU/mL) produced significant bias exceeding the 17.5% ACL threshold (Supplementary Figure S3). All recoveries remained between 92.0% and 111.5%.

#### 3.1.6. Matrix Interchangeability

Comparative analysis of CES1 concentrations across paired lithium-heparin plasma, EDTA plasma, and serum specimens (*n* = 16) confirmed complete analytical interchangeability among all matrix types. For EDTA plasma versus lithium-heparin plasma, Bland–Altman percentage analysis revealed a mean relative paired bias of −4.0% (absolute bias: −40.16 pg/mL; 95% LoA: −27.7% to 19.6%), and Passing–Bablok regression yielded a slope of 1.02 (95% CI: 0.88 to 1.13) and an intercept of −43.38 pg/mL (95% CI: −151.34 to 70.48), confirming the absence of constant or proportional bias (Supplementary Figure S4A,D). Serum measurements similarly showed excellent agreement with lithium-heparin plasma, exhibiting a mean relative paired bias of −4.8% (absolute bias: −25.12 pg/mL; 95% LoA: −27.6% to 18.0%) by Bland–Altman analysis and a Passing–Bablok slope of 1.05 (95% CI: 0.90 to 1.13) with an intercept of −58.31 pg/mL (95% CI: −185.55 to 41.42) (Supplementary Figure S4B,E). Direct comparison between serum and EDTA plasma further confirmed interchangeability, demonstrating a mean relative paired bias of +0.8% (absolute bias: −15.04 pg/mL; 95% LoA: −26.3% to 27.8%), a Passing–Bablok slope of 1.02 (95% CI: 0.93 to 1.15), and an intercept of −48.60 pg/mL (95% CI: −128.91 to 47.91) (Supplementary Figure S4C,F). Mean relative biases across all pairwise matrix comparisons remained under 5%, confirming the analytical interchangeability across all evaluated tube types.

#### 3.1.7. Sample Stability

Repeated freeze–thaw cycles up to 7 cycles produced fluctuations within the ACL (17.5%) across all concentration levels, indicating preserved assay performance (Supplementary Figure S5A). Short-term refrigerated storage at 4 °C resulted in only minor variations for up to 7 days, with no consistent decline observed (Supplementary Figure S5B). Room-temperature storage led to slightly larger changes; however, all deviations remained within the ACL throughout the 7-day period (Supplementary Figure S5C). Additionally, 1-month storage at −20 °C demonstrated excellent stability, with changes maintained within the ACL (Supplementary Figure S5D).

Furthermore, evaluation of long-term stability in clinical plasma samples (*n* = 10) stored at −80 °C for over 1 year demonstrated robust analyte preservation. Replicate-level re-analysis showed that all samples met the ACL (within ±17.5%), with low analytical variation (%CV) and percentage deviations remaining well within acceptable limits (Supplementary Table S4). Collectively, the CES1 measurements remained analytically stable over a range of routine storage and handling conditions.

### 3.2. Clinical Validation

Table 2 details the baseline profile of the 95 patients enrolled. The cohort had a median age of 56 years, was 48% male, and showed a median BMI of 22.3 kg/m^2^. Median AST and ALT levels were both 20 U/L, indicating largely preserved liver function. Median FIB-4 index was 1.10, and median shear-wave speed was 1.24 m/sec, suggesting that most patients had no advanced liver fibrosis. Regarding hepatic steatosis, B-mode US-based steatotic liver scores showed that a score of 0 was the most frequent (45%), with a decreasing prevalence in higher steatosis grades.

High measurement consistency of ATI was achieved across the study cohort. The median CV across 5 repeated ATI measurements was 1.6% (range, 0–5.2%), and the median IQR/median ratio was 3.2% (range, 0–10.3%). The *R*^2^ yielded a median of 0.94 (range, 0.74–0.99).

**Figure 1:**
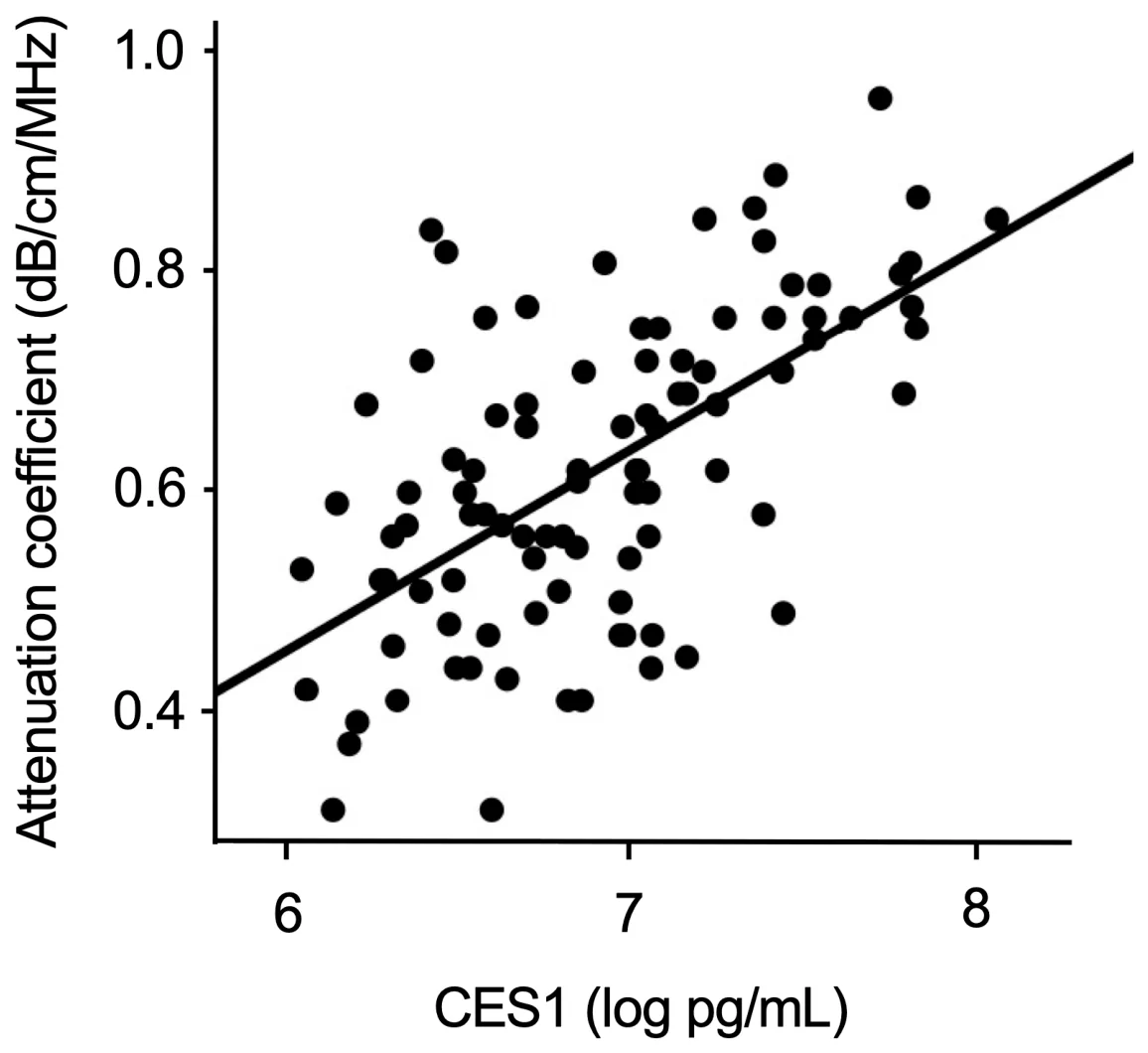
Association between plasma carboxylesterase 1 (CES1) levels and ultrasound-derived attenuation coefficient. Scatter plot illustrating the relationship between log-transformed plasma CES1 concentrations (log pg/mL) and the attenuation coefficient (dB/cm/MHz). The equation for the trend line is *y* = −0.631 + 0.183log(CES1). A significant positive correlation was identified (*r* = 0.585, *p* < 0.001). To better visualize the distribution of skewed data, specific axes are log-transformed. Note that statistical significance and correlation coefficients were determined using Spearman’s rank correlation analysis, which is independent of data transformation.

Correlation results are presented in Table 3 and Figure 1. Plasma CES1 concentration was significantly and positively correlated with BMI (*r* = 0.418, *p* < 0.001), AST (*r* = 0.341, *p* < 0.001), ALT (*r* = 0.403, *p* < 0.001), γ-GT (*r* = 0.362, *p* < 0.001), the ultrasound-based steatosis score (*r* = 0.570, *p* < 0.001), and AC (*r* = 0.585, *p* < 0.001).

**Figure 2:**
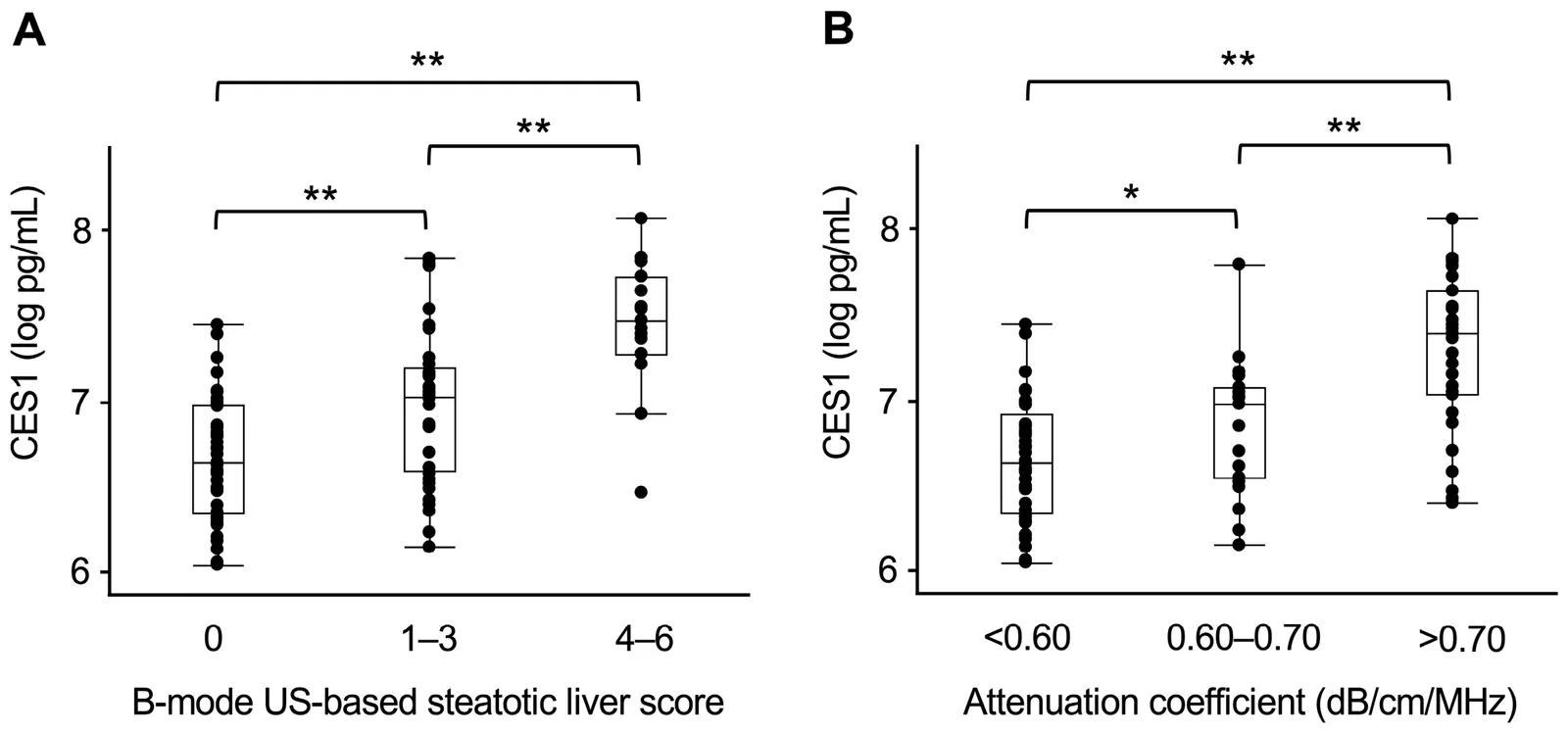
Distribution of plasma CES1 concentrations across ultrasound-defined hepatic steatosis categories. (**A**) Plasma CES1 levels categorized by B-mode ultrasound-based steatotic liver scores (Score 0, *n* = 43; Score 1–3, *n* = 37; Score 4–6, *n* = 15). (**B**) Plasma CES1 levels across exploratory attenuation coefficient (ATI) categories (<0.60 dB/cm/MHz, *n* = 41; 0.60–0.70 dB/cm/MHz [borderline], *n* = 23; >0.70 dB/cm/MHz, *n* = 31). Note that these ATI categories represent an exploratory analysis for observation of incremental trends and do not constitute established clinical stages (primary binary steatotic liver threshold = 0.66 dB/cm/MHz). Data are presented as dot plots with median and interquartile ranges. Global group differences were evaluated using the Kruskal–Wallis test (A: *H* = 28.02, *df* = 2, *p* < 0.0001, $\eta_{H}^{2}$ = 0.283; B: *H* = 31.85, *df* = 2, *p* < 0.0001, $\eta_{H}^{2}$ = 0.325). Pairwise comparisons were adjusted for multiplicity using the Steel–Dwass post hoc test (* *P*_adj_ < 0.05, ** *P*_adj_ < 0.01). Exact adjusted *p*-values and pairwise effect sizes (*r*) are reported in the text. Independent of the statistical analysis, specific axes are log-transformed to better visualize the distribution of skewed data.

To evaluate plasma CES1 distribution across varying degrees of hepatic fat accumulation, exploratory subgroup analyses were conducted. As shown in Figure 2A, plasma CES1 concentrations significantly increased with higher B-mode ultrasound H-categories (Score 0 [*n* = 43], Score 1–3 [*n* = 37], Score 4–6 [*n* = 15]; Kruskal–Wallis global *H* = 28.02, *df* = 2, *p* < 0.0001, $\eta_{H}^{2}$ = 0.283). Post hoc Steel–Dwass tests demonstrated significant stepwise differences between Score 4–6 and Score 0 (*P*_adj_ < 0.0001, *r* = 0.630), Score 4–6 and Score 1–3 (*P*_adj_ = 0.0008, *r* = 0.464), as well as Score 1–3 and Score 0 (*P*_adj_ = 0.0024, *r* = 0.340).

Furthermore, while 0.66 dB/cm/MHz served as the primary binary diagnostic cut-off for ultrasound-defined steatotic liver [28], an exploratory three-category classification based on ATI values (<0.60 dB/cm/MHz [*n* = 41], 0.60–0.70 dB/cm/MHz [borderline, *n* = 23], and ≥0.71 dB/cm/MHz [*n* = 31]) was analyzed to observe continuous trends (Figure 2B). Global Kruskal–Wallis testing revealed significant differences across the three ATI categories (*H* = 31.85, *df* = 2, *p* < 0.0001, $\eta_{H}^{2}$ = 0.325). Subsequent Steel–Dwass post hoc tests confirmed significant elevations in plasma CES1 between ≥0.71 and <0.60 dB/cm/MHz (*P*_adj_ < 0.0001, *r* = 0.623), ≥0.71 and 0.60–0.70 dB/cm/MHz (*P*_adj_ = 0.0016, *r* = 0.469), and between borderline (0.60–0.70) and <0.60 dB/cm/MHz (*P*_adj_ = 0.0388, *r* = 0.305).

**Figure 3:**
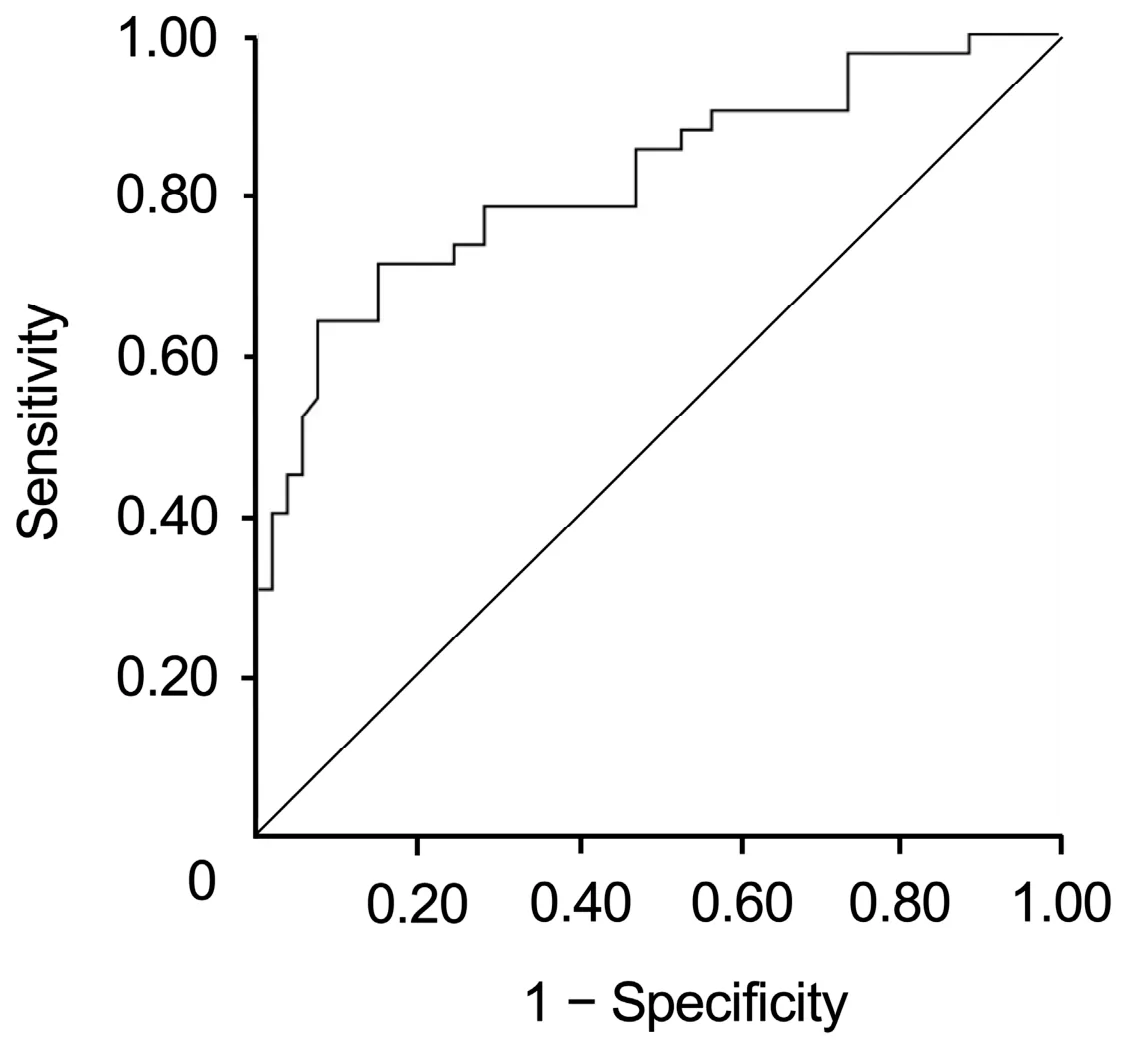
Receiver operating characteristic (ROC) curve analysis of CES1. ROC curve of CES1 for detecting steatotic liver under an attenuation coefficient cutoff of 0.66 dB/cm/MHz (steatotic group, *n* = 42; non-steatotic group, *n* = 53). The area under the curve (AUC) is 0.82 (95% confidence interval [CI]: 0.73–0.91, *p* < 0.001). At the optimal cutoff value of 1185 pg/mL determined by the Youden index, the sensitivity and specificity are 64% and 92%, respectively. The 95% CI for the AUC was estimated using a bootstrap resampling method with 2500 iterations.

ROC analysis yielded an AUC of 0.82 for CES1 in detecting steatotic liver under an AC cutoff of 0.66 dB/cm/MHz [26] (Figure 3; steatotic group, *n* = 42; non-steatotic group, *n* = 53).

The univariable and multivariable logistic regression analyses assessing factors associated with ultrasound-defined steatotic liver are presented in Table 4. In the univariable analysis, higher BMI (Crude OR: 1.35, 95% CI: 1.18–1.58, *p* < 0.001), ALT (Crude OR: 1.06, 95% CI: 1.02–1.10, *p* < 0.001), γ-GT (Crude OR: 1.01, 95% CI: 1.00–1.03, *p* = 0.021), albumin (Crude OR: 4.21, 95% CI: 1.06–18.7, *p* = 0.042), and plasma CES1 levels (Crude OR: 1.03 per 10 pg/mL increase, 95% CI: 1.02–1.05, *p* < 0.001) were significantly associated with ultrasound-defined steatotic liver.

In the multivariable logistic regression model incorporating clinically relevant covariates (BMI and γ-GT), plasma CES1 levels remained significantly associated with steatotic liver (adjusted OR: 1.03 per 10 pg/mL increase, 95% CI: 1.01–1.04, *p* < 0.001), alongside BMI (adjusted OR: 1.25, 95% CI: 1.06–1.49, *p* = 0.006). Multicollinearity was minimal (all VIFs < 5.0), and the model demonstrated acceptable calibration (Lack of Fit *p* > 0.05). Internal validation via 1000 bootstrap resamples confirmed the stability of these estimates.

## 4. Discussion

The comprehensive characterization conducted in this study supports the fully automated measurement of human CES1 as a reliable tool for clinical research and its potential clinical implementation. By adhering to standardized protocols [17], our validation confirmed that the automated system met the requisite precision and sensitivity thresholds. The assay exhibited analytical stability and consistency under various conditions, including different matrix types (blood collection tubes), dilution linearity, and recovery tests. Furthermore, our extensive stability data provide essential guidelines for specimen handling, marking a critical step toward the clinical standardization of this biomarker.

Building upon the validation framework established in our previous study on fibroblast growth factor 21 (FGF21) [18], the high precision achieved for CES1 measurement reinforces the robustness and generalizability of this automated system. Rather than merely confirming the general advantages of automation, our consecutive successful validations across different biomarkers highlight the platform’s consistent reliability for clinical applications.

While these findings establish the fundamental analytical feasibility of automated plasma CES1 measurement, several assay-level caveats remain. As circulating CES1 is a novel candidate biomarker without established international reference standards, direct trueness assessments and lot-to-lot variability remain to be evaluated. Furthermore, our evaluation of potential endogenous interferents was conducted as a preliminary screening using a single pooled plasma matrix; thus, comprehensive multi-matrix testing according to CLSI guidelines is warranted prior to broad clinical adoption.

Previous studies have reported an association between plasma/serum CES1 levels and hepatic steatosis [13,14,15], although these studies relied primarily on qualitative B-mode ultrasound. Recently, Barovic et al. [16] demonstrated a significant correlation (*r* = 0.56) between plasma CES1 levels and magnetic resonance imaging (MRI)-determined liver fat, which is a non-invasive reference standard. Consistent with these findings, we observed a similar correlation using ultrasound ATI (*r* = 0.585). Although ATI is more accessible, recent meta-analyses [28,29] have validated its diagnostic performance as comparable to that of MRI-proton density fat fraction (PDFF). To our knowledge, this is the first study to align circulating CES1 levels with ATI-derived quantitative parameters, reinforcing its potential as a precise, non-invasive surrogate marker for the degree of hepatic steatosis. A minor limitation regarding imaging assessment is that hepatic steatosis was identified using B-mode ultrasound and ATI rather than MRI-PDFF or liver biopsy, which serve as definitive reference standards. Although ultrasound-based modalities are highly accessible and suitable for routine screening, future prospective studies incorporating MRI-PDFF or biopsy-confirmed cohorts are desirable to confirm absolute diagnostic accuracy.

Among the various blood-based non-invasive tools for hepatic steatosis screening, the Fatty Liver Index (FLI) remains a primary benchmark [30], with clinical guidelines [31] and meta-analyses [32] reporting area under the curve (AUC) values generally ranging from 0.80 to 0.85 across diverse populations. In the current study, because waist circumference was not routinely measured, we could not directly calculate the FLI or perform a head-to-head comparison within our cohort. Nevertheless, plasma CES1 alone demonstrated promising diagnostic performance (AUC: 0.82) for ultrasound-defined steatotic liver. Because these data were derived from a single institution, larger, multicenter prospective studies incorporating complete anthropometric parameters (including waist circumference) are warranted to validate these findings and directly compare CES1 with established scoring systems such as the FLI.

Importantly, after adjusting for key clinical confounders—including BMI and γ-GT—using a multivariable logistic regression model with continuous predictors, plasma CES1 levels remained significantly associated with ultrasound-defined steatotic liver (adjusted OR: 1.03 per 10 pg/mL increase, 95% CI: 1.01–1.04, *p* < 0.001). This indicates that elevated circulating CES1 is linked to hepatic steatosis independently of general obesity and liver injury markers, supporting its potential utility as a non-invasive biological indicator. Combining plasma CES1 with routine clinical metrics (such as ALT, γ-GT, or BMI) or integrating it into multi-parametric models (such as the FLI), as well as with other novel circulating biomarkers such as FGF21 [18], could therefore yield additive diagnostic gains rather than redundant ones, potentially surpassing the accuracy of existing individual scoring systems and providing a more refined, accessible, and cost-effective non-imaging-based framework for screening.

Interpreting the biological relevance of circulating CES1 requires several important considerations. Given our cross-sectional design, we cannot definitively determine whether elevated plasma CES1 acts as a causal driver, a compensatory response to metabolic stress, a marker of passive release from liver injury, or a correlated signal. Functional studies indicate that hepatic CES1 critically regulates lipid metabolism; its deficiency promotes steatosis [33], whereas its FXR-mediated induction attenuates lipid accumulation [34]. However, hepatic biomarkers are broadly sensitive to inflammatory, toxic, or cholestatic stressors, where pathways like JAK1/STAT3 signaling can alter biomarker dynamics independently of steatosis [35]. As the clinical specificity of CES1 for hepatic steatosis remains unestablished, circulating levels must be interpreted cautiously. Longitudinal and mechanistic studies are needed to clarify its exact pathophysiological role.

This study has several limitations. First, our clinical evaluation is subject to spectrum restriction and sample size constraints. To approximate a general health-screening population, we intentionally excluded individuals aged ≥70 years, those with severe obesity (BMI ≥ 30 kg/m^2^), suspected advanced fibrosis (FIB-4 ≥ 2.67), and habitual drinkers. These criteria enhance internal homogeneity for early screening assessment, but they inevitably limit external validity. Specifically, advanced fibrosis with reduced functional hepatocyte mass (“burned-out steatosis”) might lower CES1 levels and increase false negatives, whereas age-related metabolic shifts, polypharmacy, or alcohol exposure could alter baseline cutoffs. Furthermore, our modest sample size (*n* = 95) from a single Japanese institution renders this a preliminary evaluation. Validation in larger, multi-center, and ethnically diverse real-world cohorts is necessary to confirm parameter stability and generalizability.

Finally, our cross-sectional design with binary ultrasound outcomes restricts our findings strictly to initial steatosis screening. Because screening requires distinct validation from disease staging [36] and therapeutic monitoring [37], longitudinal studies with serial measurements are essential to determine whether plasma CES1 can track disease progression or treatment response. In addition, detailed glycemic status, specific metabolic comorbidities, concurrent medications, and body-composition metrics (e.g., waist circumference or visceral fat) were not systematically recorded. Consequently, potential residual confounding from these unmeasured clinical variables cannot be fully excluded.

## 5. Conclusions

This study provides a comprehensive evaluation of the analytical reliability and pre-analytical stability of plasma CES1 measurements, facilitated by a fully automated immunoassay platform. Our findings demonstrated that circulating CES1 is a reproducible and clinically relevant biomarker for identifying ultrasound-defined hepatic steatosis. Although CES1 shows good accuracy as a standalone marker, its integration into other clinical algorithms holds considerable potential for advancing non-invasive, non-imaging-based diagnostic strategies. However, this preliminary evaluation was conducted in a cohort tailored to approximate a general health-screening population and is thus subject to clinical spectrum restriction. Consequently, large-scale, prospective validation studies in broader, unrestricted real-world populations are required to fully confirm its generalizability and clinical utility for widespread SLD screening.

## Figures and Tables

**Table 1:** Intra- and inter-assay precisions.

Sample	Intra-Assay Precision			Inter-Assay Precision		
	Low	Medium	High	Low	Medium	High
Number	20	20	20	25	25	25
Mean, pg/mL	207	696	2656	209	693	2651
SD, pg/mL	16.9	38.5	99.9	18.8	43.6	185
CV (%)	8.2	5.5	3.8	9.0	6.3	7.0

Abbreviations: SD = standard deviation; CV = coefficient of variation.

**Table 2:** Baseline clinical characteristics of patients (*n* = 95).

Characteristics	Reference Ranges		
Age, years		56	(27–69)
Male		46	(48)
BMI, kg/m^2^		22.3	(15.4–29.3)
AST, U/L	13–30	20	(13–67)
ALT, U/L	M: 10–42, F: 7–23	20	(7–134)
γ-GT, U/L	M: 13–64, F: 9–32	21	(6–414)
Total bilirubin, μmol/L	6.84–25.7	13.7	(5.13–47.9)
Albumin, g/L	41–51	42	(36–51)
Platelet count, ×10^9^/L	158–348	232	(139–466)
FIB-4 index		1.10	(0.40–2.28)
Shear-wave speed, m/sec		1.24	(0.94–1.58)
B-mode US-based steatotic Liver scoring system			
0		43	(45)
1		6	(6)
2		22	(23)
3		9	(9)
4		8	(8)
5		6	(6)
6		1	(1)
ATI quality parameters			
Attenuation coefficient, dB/cm/MHz		0.63	(0.32–0.97)
CV, %		1.6	(0–5.2)
IQR/Median ratio, %		3.2	(0–10.3)
*R*^2^		0.94	(0.74–0.99)
Technically invalid/rejected acquisitions,		0	(0)

Data are presented as *n* (%) or median (range). Abbreviations: BMI = body mass index; AST = aspartate aminotransferase; ALT = alanine aminotransferase; γ-GT = γ-glutamyl transpeptidase; FIB-4 = fibrosis-4; US = ultrasonography; ATI = attenuation imaging; CV = coefficient of variation; IQR = interquartile range; *R*^2^ = coefficient of determination; M = male; F = female. FIB-4 index = (AST × Age)/(Platelet count × √ALT).

**Table 3:** Correlations between clinical findings and carboxylesterase 1 (CES1) levels (*n* = 95).

Clinical Parameters	Statistics		
	*r*	(95% CIs)	*p* Value
Age, years	0.169	(−0.051–0.376)	0.102
BMI, kg/m^2^	0.418	(0.235–0.567)	<0.001
AST, U/L	0.341	(0.141–0.522)	<0.001
ALT, U/L	0.403	(0.195–0.581)	<0.001
γ-GT, U/L	0.362	(0.156–0.554)	<0.001
Total bilirubin, μmol/L	0.083	(−0.117–0.280)	0.427
Albumin, g/L	0.119	(−0.112–0.340)	0.253
Platelet count, ×10^9^/L	0.033	(−0.184–0.245)	0.749
FIB-4 index	0.118	(−0.100–0.326)	0.256
Shear-wave speed, m/sec	0.132	(−0.071–0.336)	0.202
B-mode scoring system	0.570	(0.392–0.709)	<0.001
Attenuation coefficient, dB/cm/MHz	0.585	(0.406–0.725)	<0.001

Abbreviations: *r* = correlation coefficient; CIs = confidence intervals; BMI = body mass index; AST = aspartate aminotransferase; ALT = alanine aminotransferase; γ-GT = γ-glutamyl transpeptidase; FIB-4 = fibrosis-4. FIB-4 index = (AST × Age)/(Platelet count × √ALT).

**Table 4:** Univariable and multivariable logistic regression analysis of factors associated with steatotic liver (attenuation coefficient ≥ 0.66 dB/cm/MHz; ultrasound-defined steatotic group, *n* = 42; non-steatotic group, *n* = 53).

	Crude OR	95% CIs	*p* Value	Adjusted OR	95% CIs	*p* Value
Age (years)	1.00	0.96–1.04	0.887			
Sex (Male vs. Female)	1.12	0.50–2.53	0.784			
BMI (kg/m^2^)	1.35	1.18–1.58	<0.001	1.25	1.06–1.49	0.006
ALT (U/L)	1.06	1.02–1.10	<0.001			
γ-GT (U/L)	1.01	1.00–1.03	0.021	1.00	0.99–1.02	0.664
Total bilirubin (μmol/L)	1.33	0.49–3.71	0.566			
Albumin (g/L)	4.21	1.06–18.7	0.042			
Platelet count (×10^9^/L)	1.00	0.99–1.01	0.928			
FIB-4 index	1.01	0.37–2.73	0.977			
Shear-wave speed (m/sec)	2.33	0.07–82.4	0.635			
CES1 (per 10 pg/mL increase)	1.03	1.02–1.05	<0.001	1.03	1.01–1.04	<0.001

Multivariable logistic regression analysis was performed, retaining continuous variables as continuous predictors without dichotomization. Variance inflation factors (VIFs) were all < 5.0, indicating no significant multicollinearity. Model calibration was confirmed by the Lack of Fit test (*p* > 0.05). Internal validation was performed using a 1000-sample bootstrap analysis. Abbreviations: OR = odds ratio; CIs = confidence intervals; BMI = body mass index; ALT = alanine aminotransferase; γ-GT = γ-glutamyl transpeptidase; FIB-4 = fibrosis-4; CES1 = carboxylesterase 1. FIB-4 index = (AST ×Age)/(Platelet count ×√ALT).

## Data Availability

The data presented in this study are available on request from the corresponding author due to privacy and ethical restrictions.

## Supplementary Materials

The following supporting information can be downloaded at https://www.mdpi.com/article/10.3390/diagnostics16162658/s1. Table S1: B-mode ultrasound-based steatotic liver scoring system; Table S2: Dilution linearity of CES1 assay; Table S3: Recovery rate of each sample in the spike recovery tests; Table S4: Long-term stability of human plasma CES1 stored at −80 °C for over 1 year; Figure S1: Participant flow diagram; Figure S2: Hepatic steatosis assessment via ultrasound attenuation imaging (ATI); Figure S3: Assessment of endogenous interferents in plasma carboxylesterase 1 (CES1) immunoassay; Figure S4: Method comparison and interchangeability of plasma CES1 quantification across diverse blood collection matrices; Figure S5: Analytical stability of plasma CES1 under diverse storage and handling conditions.

## Abbreviations

AC	attenuation coefficient
ACL	acceptable change limit
ALT	alanine aminotransferase
AST	aspartate aminotransferase
ATI	attenuation imaging
AUC	area under the curve
BMI	body mass index
CES1	carboxylesterase 1
CI	confidence interval
CV	coefficient of variation
EDTA	ethylenediaminetetraacetic acid
ELISA	enzyme-linked immunosorbent assay
EPV	events per variable
FGF21	fibroblast growth factor 21
FIB-4	fibrosis-4
FLI	fatty liver index
γ-GT	γ-glutamyl transpeptidase
LLOQ	lower limit of quantification
NAFLD	nonalcoholic fatty liver disease
MASLD	metabolic dysfunction-associated steatotic liver disease
MRI	magnetic resonance imaging
m-ROI	measurement region of interest
OR	odds ratio
PDFF	proton density fat fraction
RF	rheumatoid factor
ROC	receiver operating characteristic
SD	standard deviation
SLD	steatotic liver disease
SWE	shear-wave elastography
US	ultrasonography
VIFs	variance inflation factors
