# Supplementary material for: Analytical Validation and Preliminary Clinical Evaluation of Plasma Carboxylesterase 1 for Ultrasound-Defined Hepatic Steatosis
Source: Diagnostics (Basel). 2026 Aug 20;16(16):2658. doi: 10.3390/diagnostics16162658 (PMC13512586; doi:10.3390/diagnostics16162658)
Supplement: Supplementary file 1 [file diagnostics-16-02658-s001.zip › diagnostics-4489759-supplementary.pdf]

Supplementary Table S1. B-mode ultrasound-based steatotic liver scoring system

B mode findings	Score
**Bright liver and hepatorenal echo contrast (Score of A)**	
Bright liver and hepatorenal echo contrast were negative.	0
Either bright liver was positive or hepatorenal echo contrast was positive.	1
Bright liver was mild, and hepatorenal echo contrast was positive.	2
Bright liver was severe, and hepatorenal echo contrast was positive.	3
**Deep attenuation (Score of B)**	
Deep attenuation was negative.	0
Visualization of the diaphragm was obscure, but an observer could distinguish the diaphragm.	1
An observer could not distinguish the diaphragm.	2
**Vessel blurring (Score of C)**	
Vessel blurring was negative.	0
The borders of intrahepatic vessels were unclear, and the lumens of intrahepatic vessels were narrowed.	1
Sum score of A, B, and C, if score of A is >1.	
Total score is 0, if score of A is 0.	

Supplementary Table S2. Dilution linearity of CES1 assay

Dilition Factor	Expected Conc. (pg/mL)	Observed Conc. 1 (pg/mL)	Observed Conc. 2 (pg/mL)	Mean Observed Conc. (pg/mL)	CV (%)	Mean Recovery (%)	Residual 1 (pg/mL)	Residual 2 (pg/mL)
×2	6500	6748	7056	6902	3.2	106.2	−170.4	+137.5
×4	3250	3570	3509	3539	1.2	108.9	+126.4	+65.0
×8	1625	1638	1605	1621	1.4	99.8	−68.8	−102.0
×16	812.5	788.2	762.1	775.2	2.4	95.4	−49.9	−76.0
×32	406.3	416.7	406.4	411.5	1.8	101.3	+12.8	+2.5
×64	203.1	215.3	200.8	208.1	4.9	102.4	+28.6	+14.1
×128	101.6	114.3	91.2	102.7	15.9	101.1	+36.1	+13.0
×256	50.8	41.9	52.5	47.2	15.9	92.9	+18.0	+28.6
×512	25.4	29.1	12.2	20.7	57.9	81.4	N/A	N/A

Regression statistics (calculated for the 2-fold to 256-fold range based on replicate-level raw data, n = 16): Slope = 1.069 (95% CI: 1.050 to 1.088); Intercept = −30.4 (95% CI: −82.1 to 21.3); R^2 = 0.999; *P* value for non-linearity test (F-test) > 0.05. Residuals are defined as the difference between the observed concentration and the predicted concentration from the linear regression model. Data for the 512-fold dilution was excluded from the linear regression analysis as it falls outside the validated quantitative range due to low-concentration imprecision.

Abbreviations: CES1 = carboxylesterase 1; CV = coefficient of variation; Conc. = concentration.

Supplementary Table S3. Recovery rate of each sample in the spike recovery tests

	Spike	Measured Conc. (pg/mL)	CV (%)	Theoretical Conc. (pg/mL)	Recovery Rate (%)
Sample 1	0 pg	1234	1.4	–	–
	40 pg	1624	0.6	1634	97.6
	80 pg	1977	0.4	2034	92.9
	100 pg	2118	0.2	2234	88.4
Sample 2	0 pg	1478	0.2	–	–
	40 pg	1866	0.1	1878	97.0
	80 pg	2245	0.2	2278	95.9
	100 pg	2378	0.4	2478	90.0
Sample 3	0 pg	1761	0.7	–	–
	40 pg	2193	0.2	2161	107.8
	80 pg	2554	0.3	2561	99.1
	100 pg	2705	0.2	2761	94.4

Abbreviations: Conc. = concentration; CV = coefficient of variation.

Supplementary Table S4. Long-term stability of human plasma CES1 stored at −80°C for over 1 year

ID	Storage Duration (months)	Baseline Measurement (pg/mL)	Re-test Measurement (pg/mL)		Mean ± SD	CV (%)	Deviation from Baseline (%)
			Rep 1	Rep 2			
1	22	428.0	411.3	400.6	405.9 ± 7.61	1.9	−5.2
2	22	671.9	654.2	661.1	657.7 ± 4.89	1.2	−2.1
3	22	804.3	805.2	800.0	802.4 ± 3.89	0.7	−0.2
4	22	1879	1924	1899	1912 ± 17.2	1.1	+1.7
5	22	1447	1355	1343	1349 ± 8.75	0.5	−6.8
6	22	756.6	774.5	762.3	768.4 ± 8.58	0.2	+1.6
7	22	1097	1055	1052	1054 ± 2.34	0.6	−3.9
8	22	3196	2946	2977	2962 ± 22.2	1.7	−7.3
9	23	599.1	604.3	614.8	609.6 ± 7.45	0.9	+1.7
10	23	1678	1686	1644	1665 ± 29.1	0.8	−0.8

Rep 1 / Rep 2: Individual assay values for duplicate measurements.

%CV (Coefficient of Variation): Reflects analytical variation within each assay run (SD/Mean × 100).

% Deviation from Baseline: Calculated as (Re-test Mean – Baseline Mean) / Baseline Mean × 100

Acceptance Criteria: Stability is considered acceptable if the mean % deviation is within the acceptable change limit (within ±17.5%).

Abbreviations: SD = standard deviation

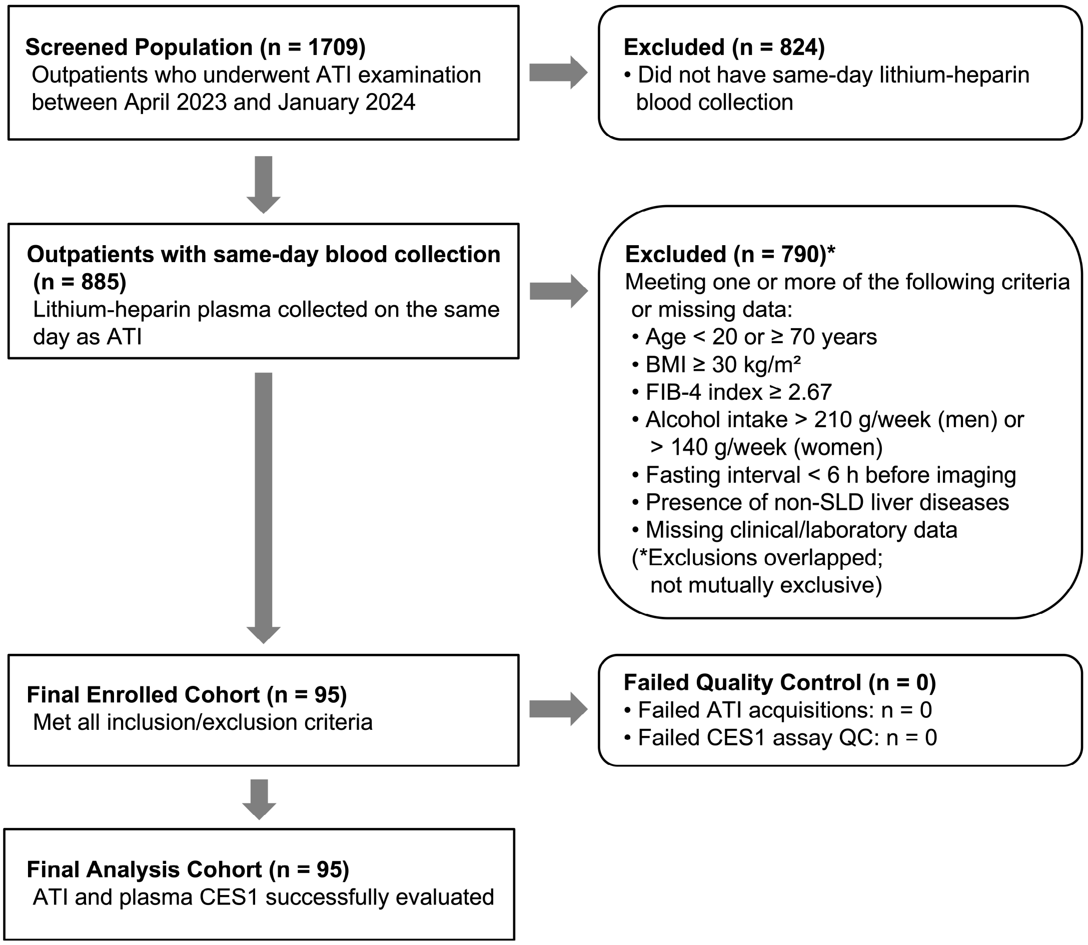

Supplementary Figure S1. Participant flow diagram

A total of 1709 consecutive outpatients who underwent ultrasound attenuation imaging (ATI) between April 2023 and January 2024 were screened. Of these, 885 patients had lithium-heparin plasma samples collected on the same day as the ATI examination. A total of 790 patients were excluded based on predefined exclusion criteria or missing clinical/laboratory data. Individual exclusion criteria were overlapping and not mutually exclusive; thus, individual exclusion counts were not separately tabulated. No participants were excluded due to quality control (QC) failure in ATI acquisitions or plasma carboxylesterase 1 (CES1) assay metrics (0% failure rate). Ultimately, 95 patients met all criteria and were included in the final analysis cohort.

Abbreviations: ATI = attenuation imaging; BMI = body mass index; CES1 = carboxylesterase 1; FIB-4 = Fibrosis-4; QC = quality control; SLD = steatotic liver disease.

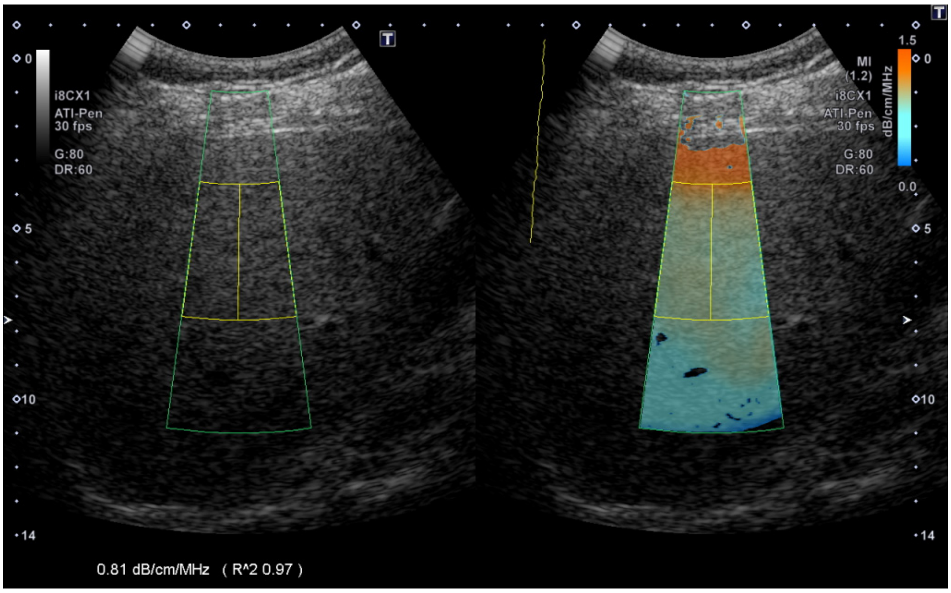

Supplementary Figure S2. Hepatic steatosis assessment via ultrasound attenuation imaging (ATI) Representative sonograms showing ATI application to evaluate liver fat content. To ensure the measurement accuracy, a fan-shaped sampling area was positioned over the liver parenchyma to exclude vascular structures and acoustic shadows. In accordance with the manufacturer's protocol, a 2 × 4 cm region of interest (delineated by the yellow line) was established, with the superior margin placed beneath the multireflection zone. Reliability of the quantification was indicated by the coefficient of determination (R^2) displayed in the lower-left corner. The system utilizes a color-coded scale to reflect data quality: red ($R^2 < 0.70$) for low quality, yellow ($R^2 = 0.70–0.90$) for acceptable quality, and white ($R^2 \geq 0.90$) for high quality. The displayed case illustrates an attenuation coefficient of 0.81 dB/cm/MHz with an R^2 value of 0.97.

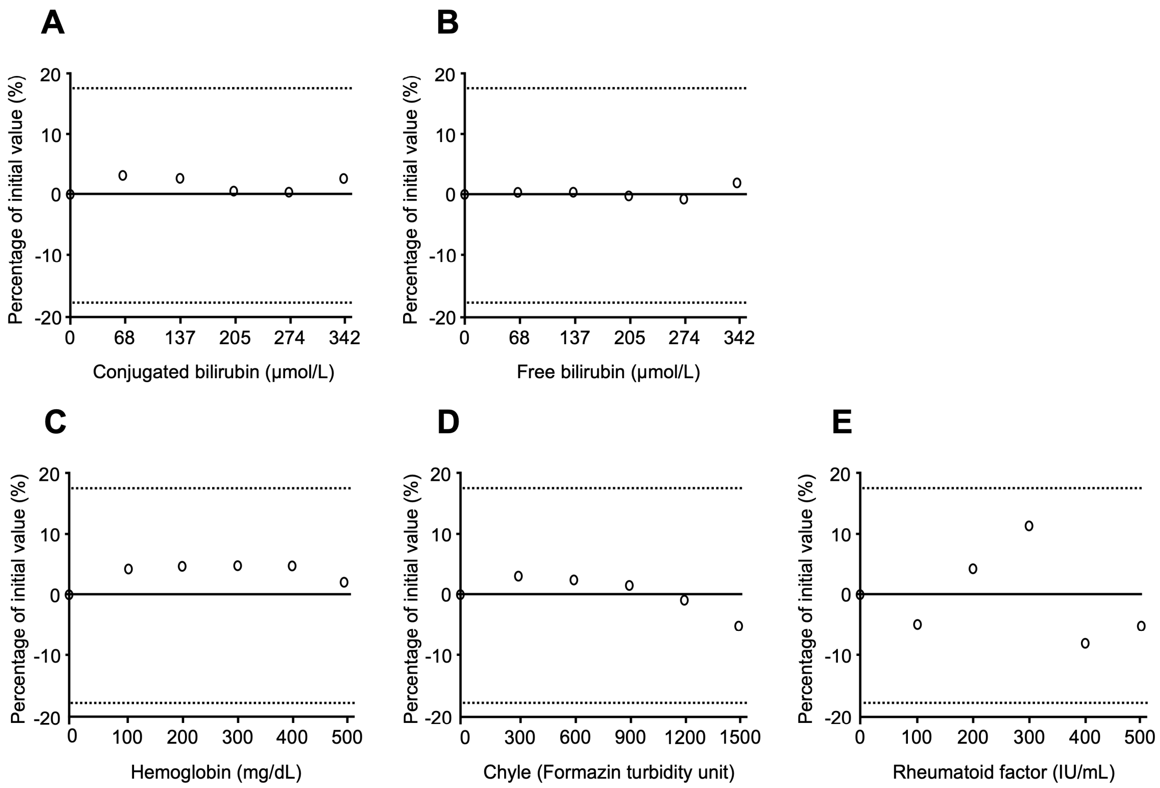

Supplementary Figure S3. Assessment of endogenous interferents in plasma carboxylesterase 1 (CES1) immunoassay

Effects of (A) conjugated bilirubin, (B) free bilirubin, (C) hemoglobin, (D) chyle, and (E) rheumatoid factor on plasma CES1 measurement. For all substances, deviations remained within the acceptable change limit (17.5%), indicating reliable assay performance.

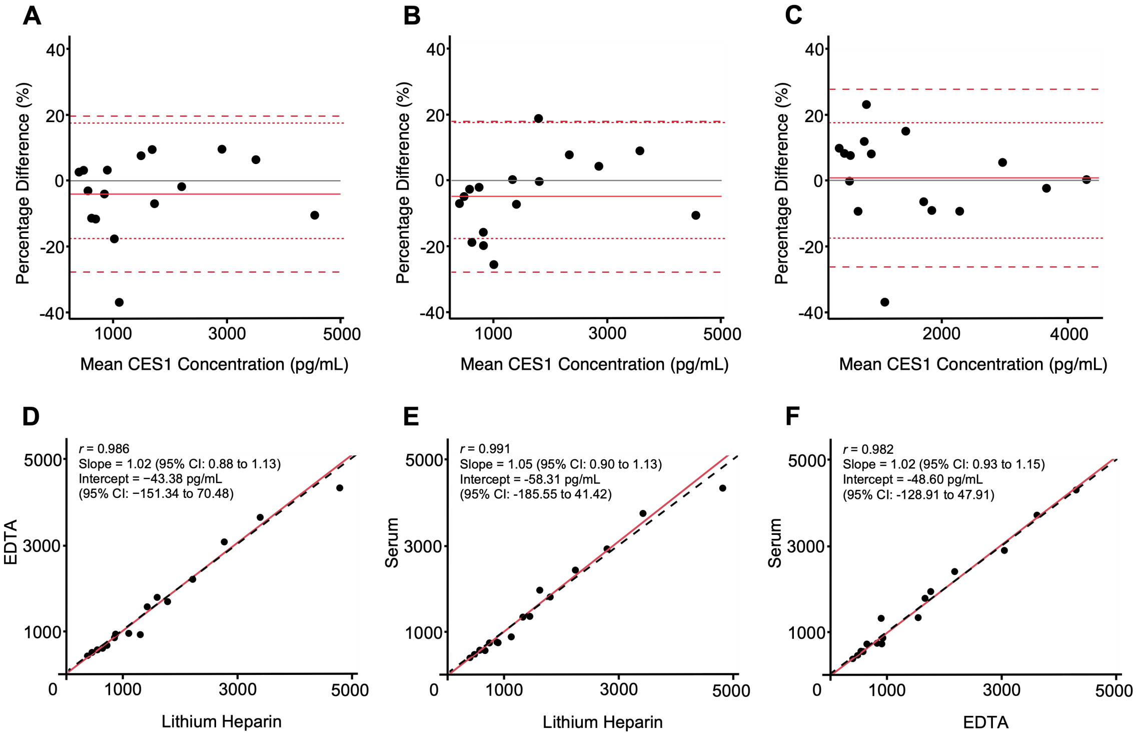

Supplementary Figure S4. Method comparison and interchangeability of plasma CES1 quantification across diverse blood collection matrices

Pairwise comparisons of CES1 concentrations among three common blood collection tube types (n = 16 paired specimens) evaluated by Bland-Altman percentage difference plots (top row; Panels A–C) and Passing-Bablok regression analysis (bottom row; Panels D–F). (A, D) EDTA plasma vs. Lithium-heparin plasma: (A) Bland-Altman plot displaying a mean relative paired bias of −4.0% (solid line) with 95% limits of agreement (LoA) from −27.7% to 19.6% (dashed lines). (D) Passing-Bablok regression plot showing a slope of 1.02 (95% CI: 0.88 to 1.13) and an intercept of −43.38 pg/mL (95% CI: −151.34 to 70.48 pg/mL). Solid line represents the regression line; dashed line indicates the line of identity (y = x). (B, E) Serum vs. Lithium-heparin plasma: (B) Bland-Altman plot demonstrating a mean relative paired bias of −4.8% (95% LoA: −27.6% to 18.0%). (E) Passing-Bablok regression plot showing a slope of 1.05 (95% CI: 0.90 to 1.13) and an intercept of −58.31 pg/mL (95% CI: −185.55 to 41.42 pg/mL). (C, F) Serum vs. EDTA plasma: (C) Bland-

Altman plot showing a mean relative paired bias of +0.8% (95% LoA: −26.3% to 27.8%). (F) Passing-Bablok regression plot showing a slope of 1.02 (95% CI: 0.93 to 1.15) and an intercept of −48.60 pg/mL (95% CI: −128.91 to 47.91 pg/mL). Horizontal dotted lines in Bland-Altman plots represent the predefined acceptable change limit (ACL, ±17.5%). All paired relative biases were well within acceptable limits, confirming analytical interchangeability among lithium-heparin plasma, EDTA plasma, and serum.

Abbreviations: CES1 = carboxylesterase 1; CI = confidence interval; EDTA = ethylenediaminetetraacetic acid; LoA = limits of agreement.

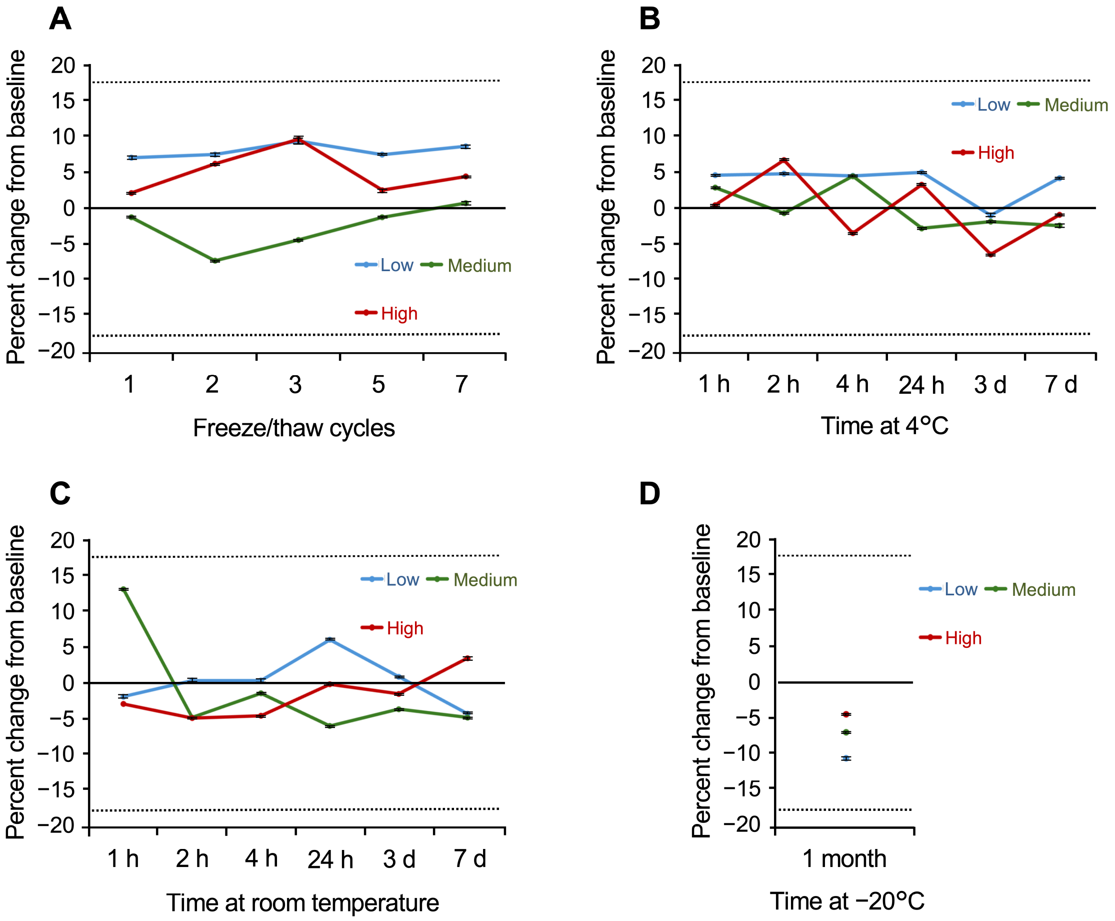

Supplementary Figure S5. Analytical stability of plasma CES1 under diverse storage and handling conditions

Percentage deviations from baseline values were evaluated across three concentration levels (low, medium, and high). For each condition and time point, a single specimen was assayed in duplicate, and data points represent the mean ± standard deviation (SD). (A) Effects of repeated freeze-thaw cycles (up to 7). Stability at (B) 4°C and (C) room temperature for durations ranging between 1 h to 7 days. (D) 1-month stability during storage at −20°C. Across all tested scenarios, measurements remained within the predefined acceptable change limit (17.5%), demonstrating the robust stability of CES1 in typical clinical and laboratory environments.
